# Bolstering the pipeline for primary care: a proposal from stakeholders in medical education

**DOI:** 10.3402/meo.v21.32146

**Published:** 2016-07-05

**Authors:** Hanyuan Shi, Kevin C. Lee

**Affiliations:** 1 Vanderbilt University Hospital, Vanderbilt University School of Medicine, Nashville, TN, USA; 2 College of Dental Medicine, Columbia University, New York, NY, USA

**Keywords:** curriculum, family medicine, residency, wellness, disparities

## Abstract

The Association of American Medical Colleges reports an impending shortage of over 90,000 primary care physicians by the year 2025. An aging and increasingly insured population demands a larger provider workforce. Unfortunately, the supply of US-trained medical students entering primary care residencies is also dwindling, and without a redesign in this country's undergraduate and graduate medical education structure, there will be significant problems in the coming decades. As an institution producing fewer and fewer trainees in primary care for one of the poorest states in the United States, we propose this curriculum to tackle the issue of the national primary care physician shortage. The aim is to promote more recruitment of medical students into family medicine through an integrated 3-year medical school education and a direct entry into a local or state primary care residency without compromising clinical experience. Using the national primary care deficit figures, we calculated that each state medical school should reserve 20–30 primary care (family medicine) residency spots, allowing students to bypass the traditional match after successfully completing a series of rigorous externships, pre-internships, core clerkships, and board exams. Robust support, advising, and personal mentoring are also incorporated to ensure adequate preparation of students. The nation's health is at risk. With full implementation in allopathic medical schools in 50 states, we propose a long-term solution that will serve to provide more than 1,000–2,700 new primary care providers annually. Ultimately, we will produce happy, experienced, and empathetic doctors to advance our nation's primary care system.

The healthcare system of the United States faces extraordinary problems. Compared with other Western nations, the US population has a shorter lifespan and a poorer overall health despite spending the most on healthcare per capita (1, 2). Some of these disparities in care stem from an unstable allocation of family medicine providers, who are overworked and unable to cover underserved areas. More worrisome, the demand for primary care physicians continues to far outstrip the dwindling supply. Aspiring clinicians face significant uncertainty and mounting pressure to choose specialist career paths in the face of rising student debt and length of training.

Positions in primary care residency programs (family medicine, internal medicine, pediatrics, medicine-pediatrics, and designated primary care medicine and pediatrics) have slowly grown each year since 2013; however, the biggest growth was in internal medicine (+254 spots from 2015) and not necessarily the designated primary care specialties (−8 spots from 2015). One of the most pervasive problems in the US healthcare system is specialty maldistribution and the subsequent trend of US medical graduates (USMGs) choosing against careers in family medicine (3). Data from the most recent 2016 National Resident Matching Program (NRMP) demonstrate a growing number of unfilled positions in family medicine programs after the main match, with 73 unfilled programs and 4.8% unfilled positions despite a 1.3% increase in spots offered. This represented a 4.0% increase in Supplemental Offer and Acceptance Program (SOAP) spots from the year before (4). More important than simply creating new positions, programs are struggling to fill these seats with qualified candidates. Designated primary care positions in internal medicine and pediatrics actually saw a decrease in available positions offered. Although 99.3% of those seats were filled by the NRMP, only 60.0% were filled by USMGs (4).Unfortunately, a paradox results: primary care and family medicine become the specialties that create the greatest health value for the nation but ultimately are viewed as the specialties that offer the least personal financial security (5).

This problem highlights the lack of accountability among US allopathic medical schools, who despite considerable public financing, have failed to produce a sustainable workforce (6). Although more students entering family medicine residencies graduate from publicly supported US MD-granting medical schools than private ones, there is still a geographic and institutional imbalance among those schools (7). Sixty-nine of the 131 US LCME-accredited medical schools (53%) in 2014 produced 80% of the graduates entering ACGME-accredited family medicine residency programs (7). Match rates were higher in publically funded allopathic medical schools (11%) than privately funded ones (7%) (8).

Only 45.3% of PGY-1 family medicine positions in 2016 were filled by USMGs (excluding osteopathic match) (5, 9). The growth in international medical graduates (IMGs) has led to their increased employment in family medicine programs (9). However, education and employment of IMGs is not the solution to the primary care shortage. Significant concerns exist about the high attrition rate (18.5% compared with 7.8% of USMGs) and abandonment of the specialty among IMGs, who left family practice 63% of the time (9). This trend threatens each program's stability if programs heavily depend on IMGs to fill their positions. Another valid concern is the quality of these graduates; many face language and cultural barriers and are unaccustomed with the US hospital and ambulatory environments.

Future of Family Medicine Project Leadership Committee recently founded task forces to determine the changes needed in the continuum of medical school education to train primary care physicians in the new landscape of US healthcare (10). They cited the physician income gap between primary care and subspecialty incomes (11) as an obstacle to physician recruitment. The Committee developed a strategic plan in response to the Patient Protection and Affordable Care Act (PPACA) calling for a well-trained primary care workforce as the sixth core tenet. Specifically, their report encouraged a redesign in the model of instruction through curricular changes in family medicine residency programs and clerkships (12). We believe that the proposal that follows can be a foundation for solving the primary care woes of the United States as outlined by the task force recommendations.

## Proposal

The challenge is immense but the solution is simple. There are 78 public allopathic MD-granting and 6 public osteopathic DO-granting medical schools in the United States (13). These state medical schools have similar missions to provide for the health of their residents and citizens. For example, the key vision of the University of Kentucky College of Medicine is to ‘improve the health of citizens of Kentucky and beyond’ (14). This naturally assumes that a proportion of the class must go into primary care–related fields, although possibly not in the state of Kentucky. Our proposal involves changes in the undergraduate medical education (UME) pathway and entrance into family medicine residencies approved by the Accreditation Council for Graduate Medical Education (ACGME). The ultimate goal is to encourage and increase the number of US medical students committed to entering a family medicine profession.

Our plan is to create and replace 20–30 spots in each public medical school class with integrated entry into a linked local or state hospital family medicine residency as part of an *accelerated* track. Mandating this in all 84 public institutions would produce up to 2,500 additional PGY-1 family medicine interns each year. The key is having no more than 6 years of total medical school education and internal entry into a family medicine residency. This would be implemented ideally in a 3-year medical school program but could be neatly incorporated in a traditional 4-year medical school programs as discussed later. The vision would involve an *accelerated* 3-year undergraduate medical curriculum for primary care, which has already been partially or fully adopted by a slew of MD and DO medical schools (Lake Erie, Texas Tech, NYU, Mercer University, LSU, Medical College of Wisconsin, UC Davis, and several others) (15, 16). The recruitment process ideally admits most of its 20–30 spots as part of medical school admissions before UME year 1 and secondarily allows some entry internally before UME year 2. The latter group will include *non*-*accelerated* traditional students that fill in for the dropouts that change their minds.

There must be significant consideration given to existing curricula in American public medical schools. Our proposal involves adding the *accelerated* track to these medical schools as follows, without changing the overall nature of the UME institutions. We assumed that most if not all of American medical schools follow a traditional 2 preclinical +2 clinical year or a 1.5+2 pathway. For both pathways implementing the *accelerated* track, we envision a total of 5–6 months of family medicine exposure and clinical training in the proposed UME curriculum.

**Fig. 1:**
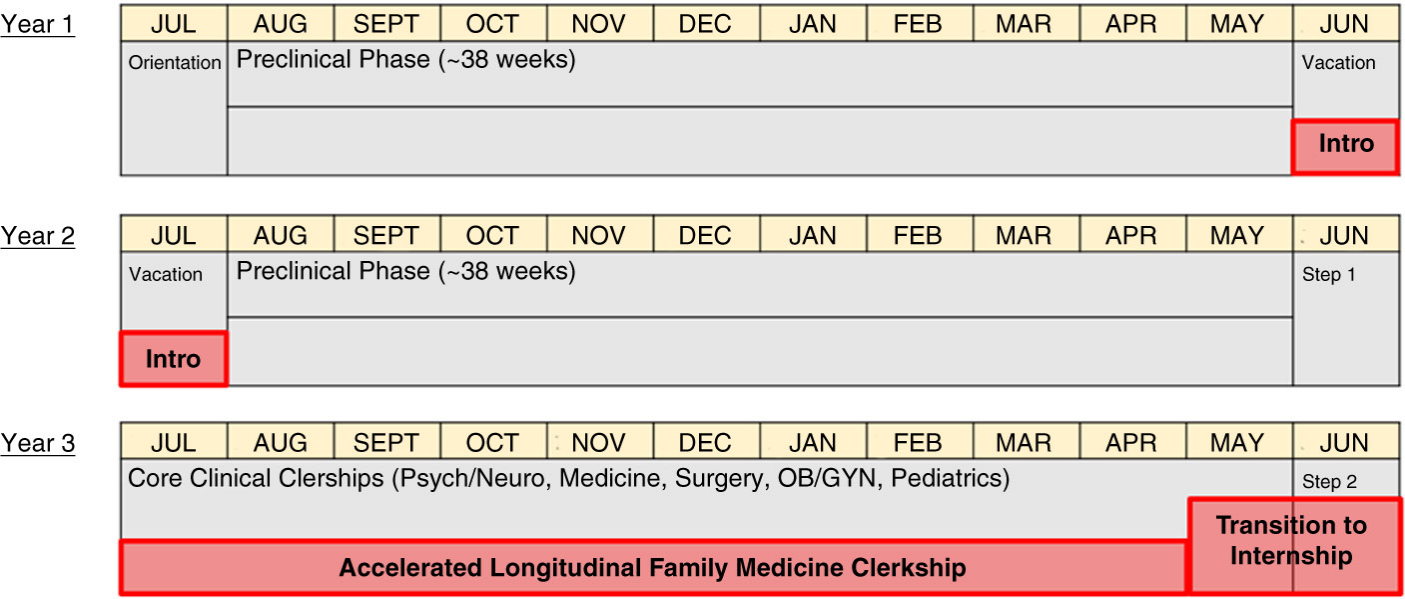
Accelerated Track in 2+2 Medical Schools. The three family medicine exposure experiences, the ‘Intro’ 2 months after UME year 1, the longitudinal clerkship in UME year 3, and a rigorous 2-month transition into internship.

Traditional 2 preclinical years+2 clinical year programs must incorporate additional family medicine training time and longitudinal experiences as shown in Fig. 1. Instead of a summer vacation, those on the *accelerated* track do 2 months of introductory foundations of family medicine (modules of family medicine principles including preventative care, common diseases, consultations in lectures and small groups) (17). These students during their clinical year then work in a longitudinal family medicine ‘continuity’ clinic in addition to their roles on the inpatient wards. Here, students follow patients over an extended period of time and begin to examine and reflect on perspectives of how chronic diseases affect lives. Finally, students participate in a capstone 2 month ‘pre-internship’ in the family medicine facility they will attend for residency. The sum of these experiences will equate to around 6 months of family medicine exposure.

**Fig. 2:**
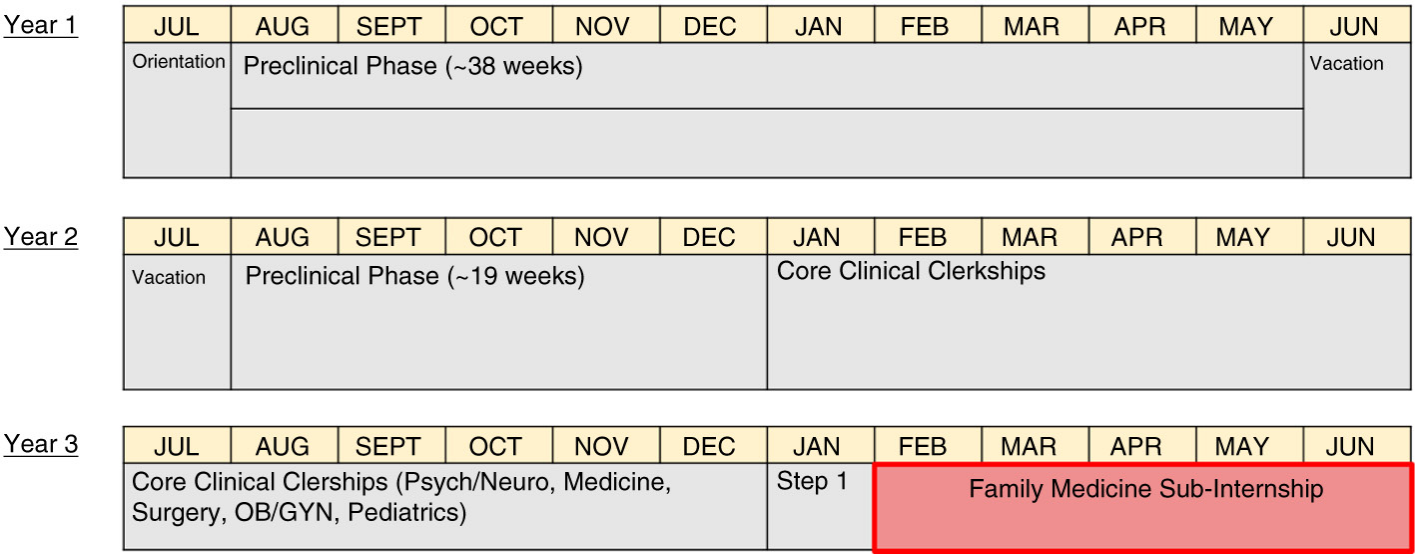
Accelerated Track in 1.5+2 Medical Schools. The 5–6 month rigorous sub-internship for students at the end of UME year 3 that reflects internship level training.

Hybrid 1.5 preclinical+2 clinical year programs can incorporate an extended family medicine pre-internship in the last half year that ranges from 5-6 months as demonstrated in Fig. 2. These pre-internships periods (effectively an extended sub-internship) would also guide students through the hospital system or clinic setting (admission orders, multidisciplinary teams, and equipment) where they would be working as residents. We believe that this scheduling addresses most if not all of the standard and institution-specific curricular standards.

The most significant change is in the capstone pre-internship blocks for students before entering residency. This is envisioned to be a major part of the curriculum that has been lacking in traditional family medicine clerkships, including specialty-specific mentorship, clinical skills/procedural workshops, inter-professional education experiences, and professional development (18). Best practices for assessment must also be employed, including a diverse array of methods like multiple-choice tests, subjective clinical evaluations, objective structured clinical examinations (OSCEs), and others (19). Self-directed learning is also necessary, as students should be provided with web-based cases (20). Literature currently suggests that ambulatory clerkship sites without residents provide comparable if not better learning for medical students (21).

Efforts will require departmental investment, medicine or family medicine, to support implementation and maintenance of these experiences (22). It is also important that the medical degree–granting institution have an affiliation agreement with regional state family medicine–accredited residency programs (ACGME). Current programs in the United States are mostly in urban areas, varying in the type of training and practice setting (community hospital/academic center, rural tracks) (23). Board-eligible single programs (not combined) are 3 years in length with opportunities for fellowships after licensure. Admittedly, this would also be a difficult negotiation. Petitioning federal and state governments for additional funding for family medicine research and training can be tricky but is crucial for the survival of this proposal (24).

## Discussion

Family medicine accounts for 13% of the US physician workforce but provides over a quarter of ambulatory care (25). These physicians have a broad scope of practice, ranging from care of special populations to providing intensive care (26). The concept of a 3-year undergraduate medical school is not new at all. In most European countries, the general practitioner, equivalent form of a family medicine physician, typically must finish a 3+3 program (first for the medical degree and second for the family medicine fellowship) (27). For the United Kingdom, students enter out of high school into a 5–6 year program where they receive a Bachelor of Medicine, Bachelor of Surgery (MBBS) degree. Here in North America, McMaster University in Ontario, Canada, first established their 3-year medical school program in 1972 and has been touted as one of the premier programs with exceptional graduates practicing across the world. Three-year accelerated programs in the United States have had success and are beginning to bud out into the public eye; the AAMC formed an eight medical school consortium in 2015 to address common goals and issues of their accelerated programs (28).

Even through this clear need, there exists the obstacle of student perception that deters many away from the field. Many students are not even exposed to family medicine, as some schools do not have a required third-year clerkship (29). Family medicine and primary care are well regarded by the lay public. More so, aspects of good medical lifestyle, societal orientation, meaningful relationships with patients and communities all are attractive to medical trainees. However, students are driven away from family medicine as an allopathic specialty because it often is the most ‘bashed’ specialty by faculty and residents of other departments (30). Medical students also cited that negative remarks about the field, negative experiences, comments that family medicine does not have academic rigor skewed their understanding of the profession. The *accelerated plan* can help students dispel these negative stereotypes from earlier specialty exposure, which has been shown in the literature to give students a greater respect for family practice (31). More so, the perception of insurmountable debt can steer away pre-medical students from medicine altogether, believing that there are no financial safety nets for young doctors (32). This proposal will solve these problems. A national 3-year pathway will target family medicine as a serious and comprehensive specialty, which will underline the role it plays in delivering acute, chronic, and a range of critical preventative medical care services in the United States.

### Strengths

Our plan alleviates many of the concerns mentioned above, including but not limited to relieving financial stress and debt, increasing connectivity, covering all pertinent parts of a traditional curriculum in less time, and ultimately delivering a well-trained supply of intelligent primary care physician (PCPs) to the US workforce.

Three years of medical school without a salary is less expensive than the traditional 4-year program in the United States. Fifty-eight percent of graduating family medicine residents already have more than $150,000 of debt and 26% have more than $250,000 of debt on completing their program (33). Even in the National Health Service Corps, young physicians often have to use extended repayment plans to balance their finances (5). Family medicine practices also suffer from unbalanced Medicare reimbursement policies, which tend to overwhelmingly favor specialists (34). In our proposal, accelerated students pay for 3-years of in-state tuition and are only responsible for the fourth if they opt out of the program. The year saved can be envisioned as an extra year of practice, extra year for research, or another year for a second degree. This allows the students to customize their UME if desired and reverses the trend of physician age creep by increasing the number of practicing physician years.

Connectivity is also a benefit to continuing work at an internal institution (35). The transition from the UME to graduate medical education (GME) is often not smooth for new interns but can be improved with an experienced third-year medical student familiar with the medical record system, teams, and environment of his or her home hospital and clinics. The biggest problems for house staff do include a lack of standardized processes and formal training for events such as discharge care, advanced planning, patient safety, and continuity of care (36). These are definitely elements that would be incorporated into an integrated curriculum, and students would pick up these skills working in the same healthcare system. In addition, better longitudinal tracking can be done for these students for their performance evaluations, clinical skills, and rapport with patients. his fast track would allow for competency-based assessment without the student stress of standardized tests and applications for the NRMP match, and with the benefit of a 6-year portfolio.

Accelerated pathways have worked at the university-medical school level in BS/MD and BA/MD programs. A 3-year medical school curriculum would still cover all pertinent basic science and core clinical foundational skills. Existing US rural-track programs have produced capable new family medicine practitioners that have already served to minimize inequality in some of America's poorest and most vulnerable communities. This does not even include successful UME+GME programs in Canada (McMaster, Calgary) that have existed without concern for over 30 years (37).

### Limitations

Critics of accelerated approaches argue that 3 years are not enough. On the contrary, limited data proves otherwise at the moment. This design includes 'deceleration' options that allow for students to exit the track if they reconsider family medicine or felt that the 3-year program was too intense. Moreover, students may still pursue traditional family medicine and primary care pathways without losing the fourth year.

These students that enter the accelerated pathway must be already committed to the specialty, which raises the question if enough advertising can attract the number needed for entrance. Choosing family medicine is associated with medical students of an older age, being female, having a rural upbringing, experience with volunteer work, and an existing interest before entering medical school (24, 38). Students matching into family medicine residencies preferred location and work/life balance the most (39). Licensed nurse practitioners and physician assistants are also quickly entering the primary care workforce with increasing autonomy, worrying some students about the prospects of finding a job after residency. However, there will always be a role and a demand for board-certified family medicine physicians in both supervising teams and clinics and as clinician-educators (40). Overall, for most students, the benefits of entering an accelerated program with a guaranteed residency outweigh these concerns.

### Implementation

The purpose of this paper is not to detail how schools will manage their resources. Administration and curricula design experts at each institution should consider relationships with teaching hospitals and the adequacies of patient volume, teaching staff, and teaching-learning activities.

Most schools have a required primary care clerkship or elective that serves as an excellent opportunity to improve practice-based learning and educate students about family medicine (41). Unfortunately, graduating medical students often still fail to understand the competencies of family medicine doctors (42). At the author's institution, there is a primary care clerkship, organized through the Department of Medicine, where students are assigned to community preceptors. As part of this clerkship, there are no formal lectures, teaching sessions, or sponsored activities by the School of Medicine. This activity is inadequate for our proposed model. The family medicine clerkship itself should be remodeled to engage and ‘recruit involved students into the ranks of family practice’ (43). Patient-centered care facilitates the most important learning moments of a family medicine clerkship and should be the focus of student clerkship experience with adjunctive educational sessions and formal lectures organized around patient care experiences (44).

By lengthening these redesigned clerkships, we hope to increase the likelihood of students choosing a career in family medicine (8).

### Future

Sustainability of this proposed change is tough. The redesign of the family medicine system must be guided by financial acumen from stakeholders and the government, must adapt best evidence, and must keep trainees happy and satisfied (45). In a changing US demographic, relatively little family medicine training occurs in actual rural or community-based settings, where a significant percentage of the population remains underserved (40). Yet, the most important aspect of this proposal is addressing the primary care physician shortage, which it will accomplish.

## Conclusions

The nation's health is at risk. The diminishing supply of US-trained family practitioners is primarily spurred by student concerns about debt, future salary, and the future of the profession. The authors propose an innovative long-term framework to these issues by training experienced, comprehensive, and passionate PCPs, to expand a needed primary care workforce in the United States. Our solution would generate an additional 1,000 to 2,500 residents each year entering the sparsely represented fields of family medicine and primary care, and would be sustained by continued efforts between UME and GME.
